# Identification and diversity of tropical maize inbred lines with resistance to common rust (*Puccinia sorghi* Schwein)

**DOI:** 10.1002/csc2.20345

**Published:** 2020-11-11

**Authors:** Julius Pyton Sserumaga, Dan Makumbi, Silvano O. Assanga, Edna K. Mageto, Susan G. Njeri, Bright M. Jumbo, Anani Y. Bruce

**Affiliations:** ^1^ National Agricultural Research Organization (NARO) National Livestock Resources Research Institute (NaLIRRI) P.O. Box 5704 Kampala Uganda; ^2^ International Maize and Wheat Improvement Center (CIMMYT) P.O. Box 1041‐00621 Nairobi Kenya; ^3^ Bayer Crop Science 1506 Hwy 69 Suite 100 Waco NE 68460 USA; ^4^ AgReliant Genetics LLC 1249 South Avenue Boone IA 50036 USA; ^5^ Crop Science Division Bayer East Africa Ltd. P.O. Box 30321‐00100 Nairobi Kenya

## Abstract

Common rust (CR) caused by *Puccinia sorghi* Schwein is one of the major foliar diseases of maize (*Zea mays* L.) in Eastern and Southern Africa. This study was conducted to (i) evaluate the response of elite tropical adapted maize inbred lines to *Puccinia sorghi* and identify resistant lines (ii) examine associations between CR disease parameters and agronomic traits, and (iii) assess the genetic diversity of the inbred lines. Fifty inbred lines were evaluated in field trials for three seasons (2017–2019) in Uganda under artificial inoculation. Disease severity was rated on a 1–9 scale at 21 (Rust 1), 28 (Rust 2), and 35 (Rust 3) days after inoculation. Area under disease progress curve (AUDPC) was calculated. The genetic diversity of the lines was assessed using 44,975 single nucleotide polymorphism markers. Combined ANOVA across seasons showed significant (*P* < .001) line mean squares for the three rust scores and AUDPC. Heritability was high for Rust 2 (0.90), Rust 3 (0.83), and AUDPC (0.93). Of the 50 lines, 12 were highly resistant to CR. Inbred lines CKL1522, CKL05010, and CKL05017 had significantly lower Rust 3 scores and AUDPC compared to the resistant check CML444 and are potential donors of CR resistance alleles. The genetic correlations between CR disease resistance parameters were positive and strong. A neighbor‐joining (NJ) tree and STRUCTURE suggested the presence of three major groups among the lines, with lines highly resistant to CR spread across the three groups. The genetic diversity among the highly resistant lines can be exploited by recycling genetically distant lines to develop new multiple disease resistant inbred lines for hybrid development and deployment.

**Abbreviations**
AD: days to anthesis
CIMMYT: International Maize and Wheat Improvement Center
EA: ear aspect
EH: ear height
ESA: eastern and southern Africa
GLS: gray leaf spot
IITA: International Institute of Tropical Agriculture
MSV: maize streak virus
NCLB: northern corn leaf blight
PA: plant aspect
PH: plant height
SD: days to silking
SSA: sub‐Saharan Africa.

## 1 INTRODUCTION

Maize (*Zea mays* L.) is grown on approximately 18.9 million hectares, with production of 41.8 million tons in eastern and southern Africa (ESA) (FAOSTAT, 2018). Maize is a source of both carbohydrates and protein for millions of people in ESA. Maize production in this region is affected by several biotic stresses including insect pests (Goergen, Kumar, Sankung, Togola, & Tamò, 2016; Kfir, Overholt, Khan, & Polaszek, 2002; Mugo et al., 2004), virus and foliar diseases (Kyetere et al., 1999; Mahuku et al., 2015; Marenya et al., 2018; Martin & Shepherd, 2009; Prasanna et al., 2020; Wangai et al., 2012), and parasitic weeds especially *Striga hermonthica* (Kanampiu et al., 2018). The major foliar diseases of economic importance in the mid‐altitude and highland ecologies of ESA are northern corn leaf blight (NCLB) caused by *Exserohilum turcicum* (Pass.) Leonard & Suggs (Pratt et al., 2003; Vivek et al., 2010), gray leaf spot (GLS) caused by *Cercospora zeae‐maydis* Tehon & Daniels (Asea et al., 2002; Bigirwa, Pratt, Adipala, & Lipps, 2001; Menkir & Ayodele, 2005; Ward, Stromberg, Nowell, & Nutter, 1999), and common rust caused by *Puccinia sorghi* Schwein (CABI, 2019; Fininsa & Yuen, 2001; Vivek et al., 2010).

Common rust (CR) of maize is widely distributed in temperate (Gingera, Davis, & Groth, 1994; Pataky & Tracy, 1999) and subtropical and tropical (Bekeko, 2019; Darino et al., 2016; Pratt & Gordon, 2006; Vivek et al., 2010) maize growing regions of the world. *Puccinia sorghi* is an obligate parasite (Anderson, Tyler, & Pryor, 1992; Pryor, 1994). Common rust disease epidemics are characterized with short latency periods of about five to ten days at temperatures of 15–25 °C and are more common at a relative humidity of at least 98% (Hooker, 1985; Pataky & Tracy, 1999). Common rust leads to loss of photosynthetic leaf area, chlorosis and premature leaf senescence, resulting in incomplete grain filling and poor yields, and low grain quality (Groth, Zeyen, Davis, & Christ, 1983a; Roelfs & Bushnell, 1985). Grain yield losses ranging from 12–75% have been reported for different maize genotypes (Dey et al., 2012; Groth et al., 1983a; Kim & Brewbaker, 1976; Shah & Dillard, 2006). Pataky (1987) estimated a 6.5% yield loss for every 10% of leaf area diseased by CR.

Several options to control CR have been proposed including use of fungicides (Pataky & Headrick, 1989; Wegulo, Rivera‐C, Martinson, & Nutter, 1998; Wright, Parkerb, Van Tilburg, & Hedderley, 2014), foliar fertilizer (Reuveni, Agapov, & Reuveni, 1994), biocontrol agents (Dey et al., 2013; Sartori et al., 2017) and resistant varieties (Headrick & Pataky, 1987; Pataky & Headrick, 1989; Pataky & Eastburn, 1993; Abedon & Tracy, 1998; Gingera et al., 1994). The development and deployment of resistant maize genotypes is the most ecologically friendly strategy to minimize the effects of *P. sorghi*, reduce the cost and adverse effects of fungicides, and significantly contribute to increased grain yield (Lübberstedt et al., 1998). Climate change projections have indicated that some areas of Africa will likely experience an increased risk of occurrence of both common and southern (*Puccinia polysora* Underw.) rust by 2050 (Ramirez‐Cabral, Kumar, & Shabani, 2017). Hence, to mitigate the potential risks, there is a need to introgress resistance to CR in inbred lines adapted to the different agro‐ecologies where the disease is a challenge. Both race specific (Hu & Hulbert, 1996; Hulbert, 1997; Hulbert, Lyons, & Bennetzen, 1991) and partial resistance (Gingera et al., 1994; Randle, Davis, & Groth, 1984) to CR have been reported. Several studies have identified quantitative trait loci (QTL) for resistance to CR on different chromosomes in different germplasm (Brown, Juvik, & Pataky, 2001; Kerns, Dudley, & Rufener, 1999; Lübberstedt et al., 1998; Olukolu, Tracy, Wisser, De Vries, & Balint‐Kurti, 2016; Rossi, Ruiz, Bonamico, & Balzarini, 2020; Zheng et al., 2018).

**Core Ideas**

- Twelve inbred lines highly resistant to common rust (CR) were identified in a 3‐year study.
- The highly resistant inbred lines were genetically diverse.
- The highly resistant inbred lines are potential donors of CR resistance alleles.

Sources of resistance to CR have been reported in temperate (Groth, Davis, Zeyen, & Mogen, 1983b; Kim & Brewbaker, 1976; Reid et al., 2017; Russell, Penny, Sprague, Guthrie, & Dicke, 1971) and in tropical maize. In tropical germplasm, inbred lines CML239–CML246 from the International Maize and Wheat Improvement Center were reported to be resistant (CIMMYT, 2005). However, there is limited published information on sources of resistance to CR in adapted maize lines for ESA. An earlier report indicated presence of resistance to CR among open‐pollinated varieties and an inbred line from Africa (Wilkinson & Hooker, 1968). In a recent study (Vivek et al., 2010), the response of hybrids formed from a limited number of lines adapted to the mid‐altitude ecology and evaluated under natural disease pressure was reported, with some lines exhibiting negative general combining ability effects for CR resistance. Zheng et al. (2018), evaluated an association mapping panel of 292 lines including some lines adapted to the mid‐altitudes under natural CR disease pressure and artificial inoculation in Kenya and Mexico respectively, and reported variable response to CR among some lines developed in Zimbabwe and Ethiopia. Rossi et al. (2020) evaluated some tropical lines from CIMMYT and reported six lines with low disease severity index for CR. The findings from these few studies indicate presence of native genetic resistance to CR among tropical maize germplasm sources. However, further research to identify new sources of resistance to CR in germplasm adapted to ESA is needed. The objectives of this study were to (i) evaluate the response of elite tropical adapted maize inbred lines to *Puccinia sorghi* and identify resistant lines (ii) examine associations between CR disease parameters and agronomic traits, and (iii) assess the genetic diversity of the inbred lines using high density single nucleotide polymorphism (SNP) DArTseq markers.

## 2 MATERIALS AND METHODS

### 2.1 Genetic materials

A set of 50 inbred lines developed by CIMMYT maize breeding programs in Kenya (46 lines), Zimbabwe and Colombia (two each) were used for this study (Table 1). The lines were developed through pedigree breeding from bi‐parental crosses except five lines (42 to 46) that were derived using the doubled haploid (DH) method. Selection of lines for use in populations for inbred line development was based on good agronomic traits, resistance to foliar diseases (NCLB and GLS), maize streak virus, and tolerance to some abiotic stresses (drought, low N, and acid soils). Some of these lines used in this study have been used to develop hybrids of FAO maturity classification series 500–600 that have been evaluated widely in ESA (Magorokosho, Vivek, MacRobert, & Tarekegne, 2010; Makumbi, 2011). Forty eight out of the 50 inbred lines were of mid‐altitude ESA adaptation, and intermediate to late in maturity (819–966 growing degree days, GDD). The GDDs were calculated by setting maximum daily temperature at 30 °C and minimum daily temperature at 10 °C (Cross & Zuber, 1972). Weather information (daily temperature, relative humidity) was obtained from the weather station near nursery fields at Kiboko Research Station in Kenya. Seed production of the lines was done at Kenya Agricultural and Livestock Research Organization (KALRO) Kiboko Research Station in Kenya in 2016 and 2017. Genetic purity of the lines was verified using SNP markers as described by Semagn et al. (2012a). The resistant inbred line check was CML444, while the susceptible check was CKL141373. These two inbred line checks were chosen based on previous assessment under severe natural CR infestation at a location used regularly for disease screening in Kenya (Vivek et al., 2010).

**TABLE 1:** List of 50 inbred lines used for common rust (Puccinia sorghi Schwein) screening at Namulonge, Uganda, 2017–2019

Line	Name	Pedigree	Background/Derivation and notes	Growing degree days
1	CKL05003	[CML202/CML395‐6]‐B‐B‐2‐1‐B‐B^:^4	MSV × drought tolerant (DT), tolerant to rust	949
2	CKL05006	[CML205/[EV7992#/EV8449‐SR]C1F2‐334‐1(OSU8i)‐1‐1‐Sn]‐B‐B‐2‐6‐B‐B‐B^:^4	MSV × DT	819
3	CKL05007	[CML205/CML202]‐B‐2‐1‐1‐B‐B^:^4	MSV tolerance	931
4	CKL05010	[CML395‐2/CML202]‐B‐3‐3‐3‐B‐B^:^4	MSV × DT	890
5	CKL05017	[CML387/CML390]‐B‐1‐1‐4‐B‐B^:^4	MSV tolerance	884
6	CKL05018	[CML387/CML390]‐B‐1‐2‐1‐B^:^4	MSV tolerance	891
7	CKL05019	[CML390/CML197]‐B‐B‐5‐1‐B^:^4	MSV tolerance	903
8	CKL05024	[KILIMA(ST94)‐S5:115/[M37W/ZM607#BF37SR…]]‐B‐B‐3‐5‐B‐B^:^4	MSV tolerance	860
9	CKL147	(CKL05019/(KU1403 × 1368)‐7‐2‐1‐B^:^5)‐B‐2‐1‐1‐1‐3‐B‐B‐B‐B	CIMMYT × IITA	919
10	CKL14504	ECAVL29‐2‐3‐3‐1‐1‐2‐B‐B‐B‐B	Extracted from synthetic	860
11	CKL14505	ECAVL29‐2‐3‐3‐3‐1‐1‐B‐B‐B‐B	Extracted from synthetic	860
12	CKL141015	(CKL05017/CML536)‐B‐55‐1‐2‐1‐1‐B‐B‐B‐B	Mid‐altitude × DT	896
13	CKL141025	(CKL05018/CML536)‐B‐37‐2‐1‐1‐B‐B‐B‐B‐B	Mid‐altitude × DT	884
14	CKL141291	(CKL05006/La Posta Seq C7‐F180‐3‐1‐1‐B‐B‐B‐B)‐B‐11‐2‐1‐3‐1‐B‐B‐B‐B	Mid‐altitude × DT	866
15	CKL141292	(CKL05006/La Posta Seq C7‐F180‐3‐1‐1‐B‐B‐B‐B)‐B‐11‐2‐1‐4‐2‐B‐B‐B‐B	Mid‐altitude × DT	866
16	CKL141020	(CKL05018/CML536)‐B‐37‐1‐1‐3‐2‐B‐B‐B‐B	Mid‐altitude × DT	884
17	CKL14500	([CML444/CML395//DTPWC8F31‐1‐1‐2‐2‐BB]‐4‐2‐2‐1‐1‐B^:^4/(9071xBabamgoyo)‐3‐1‐BBB)‐B‐1‐2‐3‐1‐1‐B‐B‐B‐B‐B	CIMMYT DT × IITA	949
18	CKL14501	([CML444/CML395//DTPWC8F31‐1‐1‐2‐2‐BB]‐4‐2‐2‐1‐1‐B^:^4/(9071xBabamgoyo)‐3‐1‐BBB)‐B‐1‐2‐3‐1‐2‐B‐B‐B‐B‐B	CIMMYT DT × IITA	915
19	CKL14502	([CML444/CML395//DTPWC8F31‐1‐1‐2‐2‐BB]‐4‐2‐2‐1‐1‐B^:^4/(9071xBabamgoyo)‐3‐1‐BBB)‐B‐1‐2‐3‐1‐3‐B‐B‐B‐B	CIMMYT DT × IITA	921
20	CKL1515	(CKL05006/CML489)‐B‐13‐1‐2‐B‐B‐B‐B‐B	Mid‐altitude × DT	884
21	CKL1522	(CKL05006/CML489)‐B‐13‐1‐5‐4‐B‐B‐B‐B	Mid‐altitude × DT	932
22	CKL14207	(CKL05017/INTA/INTB‐B‐41‐B‐14‐1‐B)‐B‐25‐3‐1‐8‐2‐B‐B‐B‐B‐B	Mid‐altitude × herbicide tolerance	872
23	CKL141248	(CKL05003/CIMCALI8843/S9243‐BB‐#‐B‐5‐1‐BB‐4‐1‐3‐1‐B)‐B‐11‐2‐2‐1‐3‐B‐B‐B	Mid‐altitude × acid soil tolerance (AT)	884
24	CKL141340	(CKL05007/CIMCALI8843/S9243‐BB‐#‐B‐5‐1‐BB‐4‐1‐3‐2‐B)‐B‐4‐1‐1‐1‐B‐B‐B‐B‐B	Mid‐altitude × AT	879
25	CKL141304	(CKL05007/CIMCALI8843/S9243‐BB‐#‐B‐5‐1‐BB‐4‐1‐3‐2‐B)‐B‐4‐2‐1‐1‐B‐B‐B‐B‐B	Mid‐altitude × AT	897
26	CKL141344	(CKL05007/CIMCALI8843/S9243‐BB‐#‐B‐5‐1‐BB‐4‐1‐3‐1‐B)‐B‐1‐2‐2‐1‐2‐B‐B‐B‐B	Mid‐altitude × AT	848
27	CKL141364	(CKL05024/CIMCALI8843/S9243‐BB‐#‐B‐5‐1‐BB‐2‐3‐1‐B)‐B‐6‐3‐1‐1‐B‐B‐B‐B‐B	Mid‐altitude × AT	861
28	CKL141373	(CKL05024/CIMCALI8843/S9243‐BB‐#‐B‐5‐1‐BB‐4‐1‐3‐2‐B)‐B‐20‐2‐1‐1‐B‐B‐B‐B	Mid‐altitude × AT	879
29	CKL141374	(CKL05024/CIMCALI8843/S9243‐BB‐#‐B‐5‐1‐BB‐2‐3‐4‐B)‐B‐9‐1‐1‐1‐B‐B‐B‐B‐B	Mid‐altitude × AT	932
30	CKL141388	ECAVL21‐37‐1‐3‐2‐1‐4‐B‐B‐B‐B	Extracted from synthetic	891
31	CKL141392	ECAVL21‐37‐3‐3‐1‐1‐2‐B‐B‐B‐B	Extracted from synthetic	954
32	CKL141398	ECAVL21‐71‐1‐2‐2‐1‐2‐B‐B‐B‐B	Extracted from synthetic	872
33	CKL141134	(MAS[MSR/312]‐117‐2‐2‐1‐B^:^6/ZM523A‐16‐2‐1‐1‐BBB)‐B‐4‐1‐2‐1‐1‐B‐B‐B‐B	MSV × DT	819
34	CKL15622	(POP 10‐B‐B/[(CML395/CML444)‐B‐4‐1‐3‐1‐B/CML395//DTPWC8F31‐1‐1‐2‐2]‐5‐1‐2‐2‐BBB‐B)‐B‐8‐8‐1‐3‐4‐B‐B	IITA × CIMMYT DT	837
35	CKL15636	(CIMCALI8843/S9243‐BB‐#‐B‐5‐1‐BB‐4‐1‐3‐2‐B/[CML444/CML395//DTPWC8F31‐4‐2‐1‐6]‐3‐1‐2‐1‐1‐B^:^5‐B)‐B‐2‐2‐1‐B‐1‐B‐B	AT × DT	848
36	CKL15643	(CIMCALI8843/S9243‐BB‐#‐B‐5‐1‐BB‐4‐1‐3‐2‐B/La Posta Seq C7‐F78‐2‐1‐1‐1‐B‐B‐B‐B)‐B‐2‐5‐2‐1‐1‐B‐B	AT × DT	867
37	CKL15644	(CIMCALI8843/S9243‐BB‐#‐B‐5‐1‐BB‐4‐1‐3‐2‐B/La Posta Seq C7‐F78‐2‐1‐1‐1‐B‐B‐B‐B)‐B‐2‐5‐2‐2‐1‐B‐B	AT × DT	854
38	CKL177000	(CKL05003/PHG39/CKL05003)‐B‐2‐1‐1‐B‐B‐B	Mid‐altitude × ex‐PVPA	878
39	CKL172721	(CKL05003/LH132/CKL05003)‐B‐2‐1‐1‐B‐B‐B	Mid‐altitude × ex‐PVPA	908
40	CKL177008	(CKL05006/CML269/TX130‐BBB‐4‐4‐B^:^5‐B/CKL05006)‐B‐3‐2‐4‐B‐B‐B	Mid‐altitude × temperate	884
41	CKL172735	(CKL05010/CML269/Tx114‐B‐6‐B^:^5‐B/CKL05010)‐B‐1‐2‐1‐B‐B‐B	Mid‐altitude × temperate	908
42	CKL1557	(CKL05003/CML444//CKL05003)DH3‐B‐B‐B‐B‐B	Mid‐altitude × DT	966
43	CKL15140	(CKL05003/La Posta Seq C7‐F64‐2‐6‐2‐2‐B‐B‐B)DH110‐B‐B‐B–B	Mid‐altitude × DT	937
44	CKL15193	(CKL05006/La Posta Seq C7‐F71‐1‐2‐1‐2‐B‐B‐B‐B)DH11‐B‐B‐B‐B‐B‐B‐B	Mid‐altitude × DT	849
45	CKL15194	(CKL05006/La Posta Seq C7‐F71‐1‐2‐1‐2‐B‐B‐B‐B)DH15‐B‐B‐B‐B‐B‐B‐B	Mid‐altitude × DT	866
46	CKL1537	(CKL05018/La Posta Seq C7‐F78‐2‐1‐1‐1‐B‐B‐B)DH58‐B‐B‐B‐B‐B‐B‐B‐B	Mid‐altitude × DT	872
47	CIMCAL2	CIMCALI8843/S9243‐BB‐#‐B‐5‐1‐BB‐4‐1‐3‐2‐B‐B‐B‐B	AT	855
48	CIMCAL4	CIMCALI8843/S9243‐BB‐#‐B‐5‐1‐BB‐4‐1‐3‐1‐B‐B‐B‐B	AT	842
49	CML395	CML395	DT, MSV	920
50	CML444	CML444	DT, low N, MSV, tolerance to rust	926

^a^ AT, acid soil tolerance; DT, drought tolerance; ex‐PVPA, expired Plant Variety Protection Act; MSV, maize streak virus.

### 2.2 Test location, experimental design, and field evaluation

The inbred lines were evaluated in field trials for three seasons between 2017–2019 at National Crops Resources Research Institute (NaCRRI), Namulonge, Uganda, under artificial inoculation with *Puccinia sorghi*. The soil type at Namulonge (0°36’ N, 32°36’ E; 1150 m asl) is sandy clay loam and is classified as Orthic Ferrasol. The mean annual rainfall at Namulonge is 1270 mm with a bimodal distribution (March–July and September–November). The experimental design was a 5 × 10 alpha‐lattice (Patterson & Williams, 1976) with two replications. The experimental unit was a two‐row plot, 5 m long, spaced 0.75 m apart and 0.25 m between plants in a row, resulting in a final plant population density of approximately 53,333 plants ha^−1^. Standard location specific recommended agronomic and cultural practices were followed. Weeding was carried out by hand and fertilizer applied at a rate of 77 kg N and 27 kg P ha^−1^ split over two applications at planting and for topdressing four weeks after planting, respectively. The trials were planted under rain‐fed conditions with supplemental irrigation provided as required for optimal disease development. Since the evaluation was carried during the main cropping season, conditions were conducive for disease development (Supplemental Table S1).

### 2.3 *Puccinia sorghi* inoculum preparation

Urediniospores of *Puccinia sorghi* were collected from infected maize leaves at NaCRRI, Namulonge, Uganda in 2016 and maintained on a known susceptible experimental hybrid CKH107887 grown in a screenhouse. Spores of *P*. *sorghi* were collected from infected leaves by brushing them into 100 ml of distilled water containing 0.2 ml of Tween 20 to make an aqueous suspension of *P*. *sorghi* (Reuveni et al., 1994). Spore concentration in the suspension was estimated to be 5 × 10^4^ spore ml^−1^ using a hemocytometer. The plants were inoculated once at the 4–6 leaf stage. Inoculation was carried out following the method of Olukolu et al. (2016), whereby a knapsack sprayer was used to deliver the spore suspension by spraying the whorl and upper surface of fully expanded leaves of each plant to run‐off.

### 2.4 Disease assessment and agronomic data recording

Disease severity was visually assessed by observing the symptoms on 28–34 plants in a plot. Sporulation was checked on both the adaxial and abaxial leaf surfaces. Disease severity was rated on nine‐point scale based on the percent leaf area showing pustules of *P. sorghi* where 1 = 0% of leaf surface diseased (no rust pustules or a few pustules scattered on the leaf surface), 2 = 1% of leaf surface diseased; 3 = 2% of leaf surface diseased; 4 = 3–5% of leaf surface diseased; 5 = 6–10% of leaf surface diseased; 6 = 11–20% of leaf surface diseased; 7 = 21–40% of leaf surface diseased; 8 = 41–80% of leaf surface diseased; and 9 = 81–100% of leaf surface diseased (CIMMYT, unpublished internal protocols). Disease reaction was scored thrice during crop growth. The first disease score (Rust 1) was taken 21 days after inoculation when there were discernible differences between plots for the severity of disease symptoms. The second score (Rust 2) was taken 28 days after inoculation, while the third score (Rust 3) was taken 35 days after inoculation. The three disease severity scores were used to calculate the area under disease progress curve (AUDPC) as:

$$\mathrm{AUDPC}\;=\;\sum_{i\;=\;1}^{n}\left[\frac{Y_{i}+Y_{i+1}}{2}\right]\left(T_{i+1}+T_{i}\right)$$

where *i* = time of rust severity rating, *T_i_* is the number of days after inoculation, and Y*_i_* is the rust severity (Shaner & Finney, 1977).

Additional traits measured on plot basis were days to anthesis (AD, recorded as days from planting to when 50% of the plants started to shed pollen), plant height (PH, measured in centimeters as the distance from the base of the plant to the base of the first tassel branch), ear height (EH, measured in centimeters as the distance from the base of the plant to the point of attachment of the top ear on a plant), and plant aspect (PA, scored on a scale of 1 to 5 for overall plant appearance where 1 = attractive plant type (uniform height and ear placement, and free of foliar diseases) while 5 = undesirable plant type (variable height and ear placement, and affected by foliar diseases). Reaction to GLS disease was recorded on a scale of 1–9 (same as for CR) when the crop was at the dough stage. All ears in a two‐row plot were harvested, weighed, and representative samples of ears shelled to determine percent moisture using a Dickey‐John multi‐grain moisture tester (DICKEY‐John Corporation, IL, USA). Grain yield (Mg ha^−1^) was calculated from cob weight assuming a shelling percentage of 80% and grain yield adjusted to 12.5% moisture content.

### 2.5 Genotyping

Seedlings of the 50 inbred lines were raised in a screenhouse at National Crops Resources Research Institute, Namulonge, Uganda. Leaf tissue was harvested following the leaf sampling protocol from LGC Genomics (http://www.lgcgroup.com/our-science/genomics-solutions/#.WXpE7ITyu70) using the plant sample collection kit from LGC Genomics (Middlesex, UK). DNA was extracted using ZR Plant/Seed DNA MiniPrepTM according to manufacturer's protocol and later shipped to Integrated Genotyping Sequence Support (IGSS) at Biosciences eastern and central Africa International Livestock Research Institute (BecA ‐ ILRI) Hub, Kenya for genotyping. Of the 50 inbred lines, 48 yielded good quality DNA for analysis. Extra quality check was carried out on 0.8% agarose gel electrophoresis with lambda DNA of 50 ng as a marker. DNA for each sample was diluted to a required concentration range of 50–100 ng μl^−1^ for the DArTseq genotyping platform. Filtered genotypic data (using a minor allele frequency of 0.1 and a minimum count of 80% of the sample size) from IGSS was verified using TASSEL v.5.2 software (Bradbury et al., 2007) to retain 44,975 SNP markers for further analysis.

### 2.6 Statistical analysis

Homogeneity of variance of the data was ascertained using Levene's test before the analysis of variance. Analyses of variance were performed using PROC MIXED of SAS (SAS Institute, 2011). Entries were considered fixed effects, whereas seasons were considered random effects. Each season‐year combination was considered an environment. In the across‐environment analysis of variance, genotype effects were tested for significance using the corresponding interaction with the environment as the error term, whereas the genotype × environment interaction was tested using the pooled error. To estimate variance components, all factors were considered random effects. Best linear unbiased prediction (BLUP; Piepho, Möhring, Melchinger, & Büchse, 2008) for all traits was computed using META‐R (Alvarado et al., 2020). Broad‐sense heritability for traits across environments was estimated as described by Hallauer, Carena, and Miranda Filho (2010). Cluster analysis following Ward's minimum variance (Ward, 1963) was used to group lines with similar response to artificial inoculation with *P. sorghi* using a combination of Rust 1, Rust 2, Rust 3, and AUDPC. The SAS procedures PROC CLUSTER and PROC TREE were used for cluster analysis and to generate the dendrograms, respectively.

To assess consistency of inbred line performance in different seasons, Kendall's (1962) coefficient of concordance (W statistic) was computed for *P. sorghi* severity based on ranks of line means across artificial inoculation conditions. Kendall's W statistic is calculated as:

$$W\;=\frac{12S}{p^{2}\left(n^{3}-n\right)-pT}\;$$

in which *S* is a sum‐of‐squares statistic over the row sums of ranks, n is the number of lines, p the number of locations, and T is a correction factor for tied ranks. Spearman rank correlation coefficients among rust severity scores and GLS were computed using PROC CORR of SAS (SAS Institute, 2011). Genetic correlations among traits were computed using META‐R (Alvarado et al., 2020). Sequential path analysis (Mohammadi, Prasanna, & Singh, 2003; Samonte, Wilson, & McClung, 1998) was used to examine cause and effect relationships among CR disease parameters and agronomic traits. In sequential path analysis, traits were categorized into first, second, and third order or higher depending on their contribution to the total variation in CR severity (Mohammadi et al., 2003, Samonte et al., 1998). We used sequential path analysis to avoid potential problems due to multicollinearity. Stepwise regression analysis was carried out in SPSS version 20 (IBM, 2011). In this analysis, Rust 3 was regressed on all other traits to identify first order traits. The first order traits were then regressed on other traits not among the first order traits to identify second‐order traits. The second‐order traits were then regressed on other traits not among the second‐order traits to identify third‐order traits. A trait was in first, second, or third order if it contributed significantly to variation in CR severity at *P* ≤ .05. Ordering traits into different categories allows identification of traits of primary (first‐order) and secondary (second‐order) importance, and then others. First‐order traits can be considered for use as indirect selection criteria. The standardized coefficient (*b* value) in stepwise regression analysis is equivalent to the direct path coefficient.

### 2.7 Diversity analysis

Genetic distance was computed between pairs of maize lines using identity by state method in TASSEL version 5.2 (Bradbury et al., 2007). A dendrogram was generated from the identity by state matrix using the neighbor‐joining option in TASSEL version 5.2, and the resulting tree was visualised and edited using the interactive tree of life (iTOL) tool (Letunic & Bork, 2019). The model‐based clustering approach implemented in STRUCTURE software (Pritchard, Stephens, & Donnelly, 2000) was used to analyze population structure, following the procedure described by Semagn et al. (2012b) and Sserumaga et al. (2019). Delta K was computed for each value of K using software Structure Harvester (Evanno, Regnaut, & Goudet, 2005).

## 3 RESULTS

### 3.1 Analysis of variance and heritability

Individual environment ANOVA revealed significant (*P* < .05) differences among entries for all three rust disease severity ratings and AUDPC except Rust 1 in 2018 (Supplemental Table S2). Heritability for the disease resistance parameters was high (0.72–0.92) in 2017–2019 except for Rust 1 in 2017 and 2018 when it was moderate (0.52) or low (0.22), respectively (Table 2). Combined ANOVA across three environments showed significant (*P* < .001) environment mean squares for two disease severity scores Rust 1 and 2, and significant (*P* < .001) line mean squares for the three rust scores and AUDPC (Table 3). The line × environment interaction was significant (*P* < .001) for only Rust 1. Heritability was moderate for Rust 1 (0.59) and high (0.83–0.93) for the other disease parameters (Table 4). The genotypic variance was larger than the environment (1.5 ×) and genotype × environment variance (13.6 ×) for Rust 2. Significant (*P* < .01) line mean squares for grain yield, AD, and GLS were recorded in 2017 and 2018 while significant (*P* < .01) line mean squares for PH and EH were recorded in 2019 (Supplemental Table S3). Combined ANOVA revealed significant (*P* < .01) environment and line mean squares for grain yield, AD, PH, EH, and GLS.

**TABLE 2:** Summary statistics, variance components and heritability of common rust severity scores and area under disease progress curve (AUDPC) for 50 tropical maize inbred lines evaluated under artificial inoculation with *P. sorghi* for three seasons, 2017–2019

Year	Parameter	Range	LSD_0.05_	CV	Genotypic variance	Residual variance	Heritability
2017	Rust 1	1.0‐2.9	0.9	31.5	0.11	0.21	0.52
	Rust 2	1.0‐5.5	1.3	25.9	0.54	0.42	0.72
	Rust 3	2.0‐8.9	2.5	28.9	2.53	1.53	0.77
	AUDPC ^a^	18.5‐79.4	16.7	21.9	132.23	67.18	0.80
2018	Rust 1	1.0‐2.5	0.9	35.1	0.03	0.22	0.22
	Rust 2	1.0‐6.6	1.3	26.5	1.02	0.43	0.83
	Rust 3	1.2‐8.4	2.2	20.6	3.51	1.13	0.86
	AUDPC	11.3‐82.2	19.3	24.2	166.77	89.18	0.79
2019	Rust 1	1.0‐4.3	1.4	36.1	0.71	0.53	0.73
	Rust 2	1.3‐7.6	1.1	14.2	1.60	0.30	0.91
	AUDPC	10.4‐66.1	10.3	14.7	141.46	24.93	0.92

^a^ AUDPC, area under disease progress curve; Rust 1, 2 and 3 are common rust severity scores 21, 28 and 35 days after inoculation, respectively.

**TABLE 3:** Mean squares from combined ANOVA of 50 inbred lines evaluated under artificial *P. sorghi* inoculation at Namulonge across three seasons (2017–2019)

Source	df	Rust 1	Rust 2	AUDPC^a^	df	Rust 3
Environment (E)	2	10.86:::	55.29:::	617.95	1	36.43:
Block (E × Rep)	57	0.53::	0.96:::	176.37:::	38	3.94:::
Line	49	1.12:::	4.55:::	674.17:::	49	9.39:::
Line × E	98	0.55:::	0.51	64.21	49	1.90
Residual	87	0.31	0.43	65.38	58	1.41

^:^, ^::^, ^:::^ Significant at the .05, .01, and .001 probability levels, respectively.

^a^ AUDPC, area under disease progress curve; Rust 1, 2 and 3 are common rust severity scores 21, 28 and 35 days after inoculation, respectively.

**TABLE 4:** BLUPs of common rust (Puccinia sorghi) disease severity and area under disease progress curve (AUDPC) of 50 tropical maize inbred lines evaluated under artificial inoculation across three seasons, 2017–2019

		Rust severity				
Line	Name	1	2	3	AUDPC^a^	Classification
1	CKL05003	1.0	2.1	4.2	27.8	MR^b^
2	CKL05006	1.5	2.7	6.1	37.9	MS
3	CKL05007	1.3	2.1	2.7	25.0	Highly resistant
4	CKL05010	1.0	1.6	1.6	19.6	Highly resistant
5	CKL05017	1.2	1.4	1.9	18.4	Highly resistant
6	CKL05018	1.3	2.1	3.2	26.2	Resistant
7	CKL05019	2.3	4.7	7.6	57.6	HS
8	CKL05024	1.4	3.1	6.6	38.2	Susceptible
9	CKL147	1.5	2.7	3.5	32.6	Resistant
10	CKL14504	1.5	2.4	3.9	31.9	Resistant
11	CKL14505	1.1	3.3	4.6	37.0	MR
12	CKL141015	1.0	1.6	3.5	22.0	Resistant
13	CKL141025	1.6	3.0	4.4	37.6	MR
14	CKL141291	1.7	2.4	2.9	28.8	Highly resistant
15	CKL141292	1.6	2.3	5.4	32.9	MR
16	CKL141020	1.1	2.1	2.5	24.5	Highly resistant
17	CKL14500	2.8	4.7	6.8	58.3	Susceptible
18	CKL14501	2.5	4.4	7.0	56.3	Susceptible
19	CKL14502	1.8	3.5	4.4	40.3	MR
20	CKL1515	1.1	2.2	3.6	27.4	Resistant
21	CKL1522	1.1	1.7	1.5	18.5	Highly resistant
22	CKL14207	1.0	1.8	2.8	22.9	Highly resistant
23	CKL141248	1.0	2.2	3.0	25.9	Highly resistant
24	CKL141340	1.7	3.8	5.7	46.7	MS
25	CKL141304	2.0	3.3	5.2	41.8	MR
26	CKL141344	1.0	2.7	4.4	32.6	MR
27	CKL141364	2.2	4.1	7.8	50.5	HS
28	CKL141373	3.1	6.6	8.7	76.9	HS
29	CKL141374	1.8	4.1	5.8	49.1	MS
30	CKL141388	1.8	3.1	5.6	41.5	MS
31	CKL141392	1.8	3.0	4.5	38.1	MR
32	CKL141398	1.4	1.8	2.3	23.5	Highly resistant
33	CKL141134	2.6	5.2	8.4	64.5	HS
34	CKL15622	1.4	1.9	2.9	25.0	Highly resistant
35	CKL15636	1.4	3.0	4.4	36.6	MR
36	CKL15643	1.3	2.8	4.2	33.8	MR
37	CKL15644	1.7	2.5	5.0	34.4	MR
38	CKL177000	2.3	3.2	4.3	39.5	MR
39	CKL172721	1.5	3.3	7.4	45.6	Susceptible
40	CKL177008	1.7	2.7	5.4	30.8	MR
41	CKL172735	1.0	2.4	2.7	25.8	Highly resistant
42	CKL1557	1.2	2.4	4.0	30.6	MR
43	CKL15140	1.9	3.3	6.5	44.6	MS
44	CKL15193	1.5	3.1	6.4	42.4	MS
45	CKL15194	1.7	3.2	5.0	39.8	MR
46	CKL1537	1.6	3.5	4.8	40.6	MR
47	CIMCAL2	2.5	4.1	6.2	51.4	MS
48	CIMCAL4	1.9	3.8	7.0	50.7	Susceptible
49	CML395	1.2	1.9	2.9	25.5	Highly resistant
50	CML444	1.3	2.5	4.0	30.7	MR
Range		1.0‐3.1	1.4‐6.6	1.5‐8.7	18.4‐76.9	
Mean (resistant)		1.2	1.9	2.6	23.7	
Mean (susceptible)		2.2	4.2	7.3	53.2	
LSD		0.9	0.9	2.0	9.2	
Heritability		0.59	0.90	0.83	0.93	
Genotypic variance		0.16	0.95	2.63	143.15	
Environment (E) variance		0.13	0.62	0.19	4.05	
Genotype × E variance		0.11	0.07	0.38	0.00	
Residual variance		0.34	0.41	1.36	64.47	

^a^ AUDPC, area under disease progress curve, Rust 1, 2 and 3 are common rust severity scores 21, 28 and 35 days after inoculation, respectively.

^b^ HS, highly susceptible; MR, moderately resistant; MS, moderately susceptible.

### 3.2 Performance and classification of lines

**FIGURE 1:**
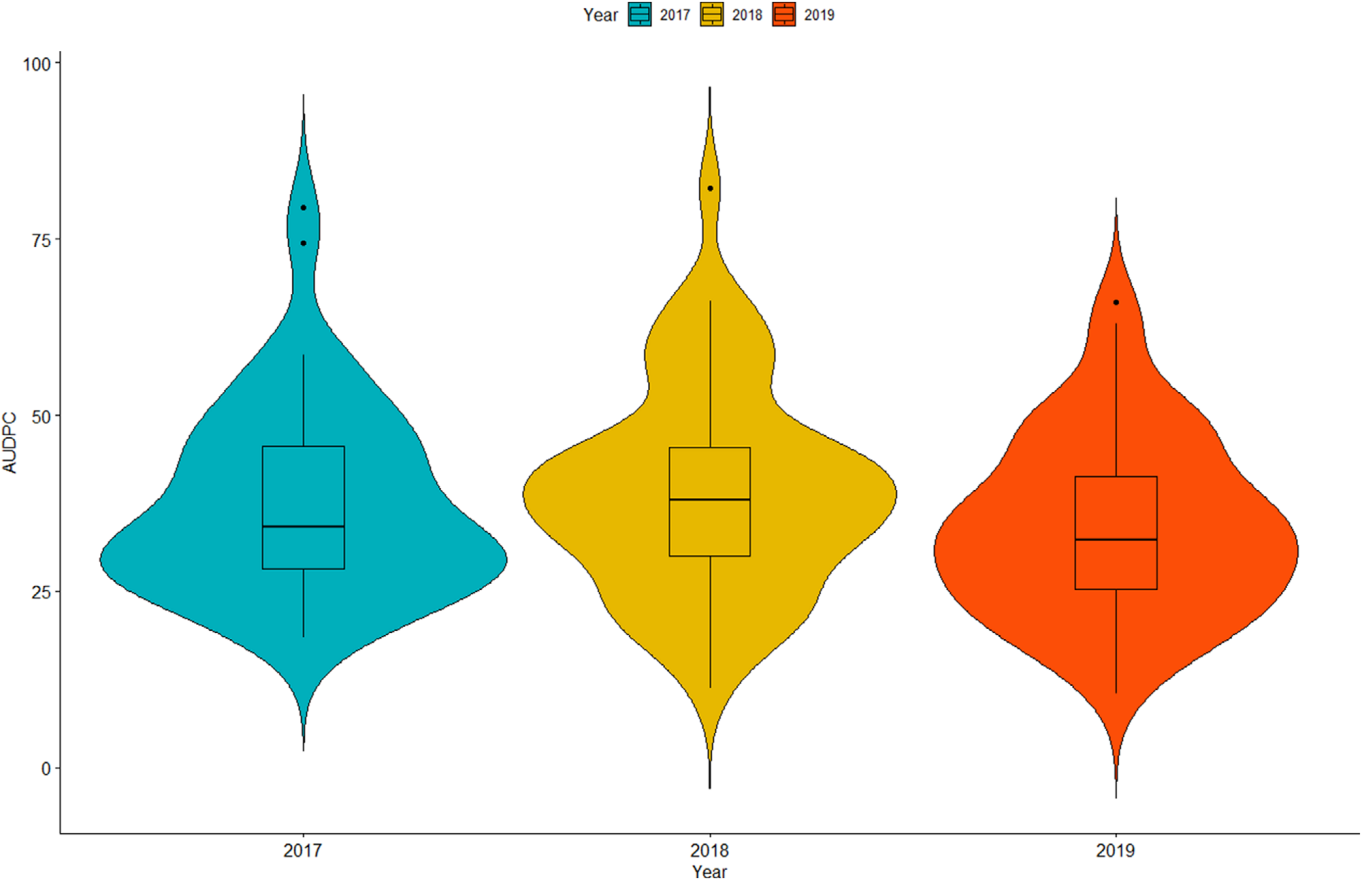
Boxplot showing distribution of AUDPC of 50 inbred lines evaluated under artificial inoculation with *P. sorghi* for three years at Namulonge, Uganda

The disease pressure was sufficient to differentiate among entries for their response to inoculation with *P. sorghi*. Disease severity scores in 2017 ranged from 1.0 to 2.9, 1.0 to 5.5 and 2.0 to 8.9 for Rust 1, Rust 2, and Rust 3, respectively (Table 2). In 2018, Rust 1 had a smaller range (1.0–2.5) compared to 2017 but a larger range was recorded in 2019 (1.0–4.3). The second Rust score varied more among entries in 2019 compared to 2018. The AUDPC ranged from 18.5 to 79.4 and 11.3 to 82.2 in 2017 and 2018, respectively (Table 2; Figure 1). In 2019 when only two disease scores were taken (Rust 1 and Rust 2), the range of AUDPC was smaller compared to the other two seasons. Across environments, disease severity scores ranged from 1.0 to 3.1, 1.4 to 6.6 and 1.5 to 8.7 for Rust 1, Rust 2, and Rust 3, respectively (Table 4). Lines showing the lowest scores (< 2) for Rust 2 were CKL05010, CKL05017, CKL141015, CKL14207, CKL141398, CKL1522, CKL15622 and CML395. Lines CKL05010, CKL05017, and CKL1522 maintained a score of <2 for Rust 3. The AUDPC varied from 18.4 to 76.9. The top three inbred lines with a score of < 2 for Rust 3 (CKL05017, CKL1522 and CKL05010) recorded 40.1%, 36.2% and 39.7% lower AUDPC, respectively, compared to the resistant check CML444. These three top lines recorded 74.5%, 76.1% and 75.9% lower AUDPC, respectively, compared to the susceptible check CKL141373.

**FIGURE 2:**
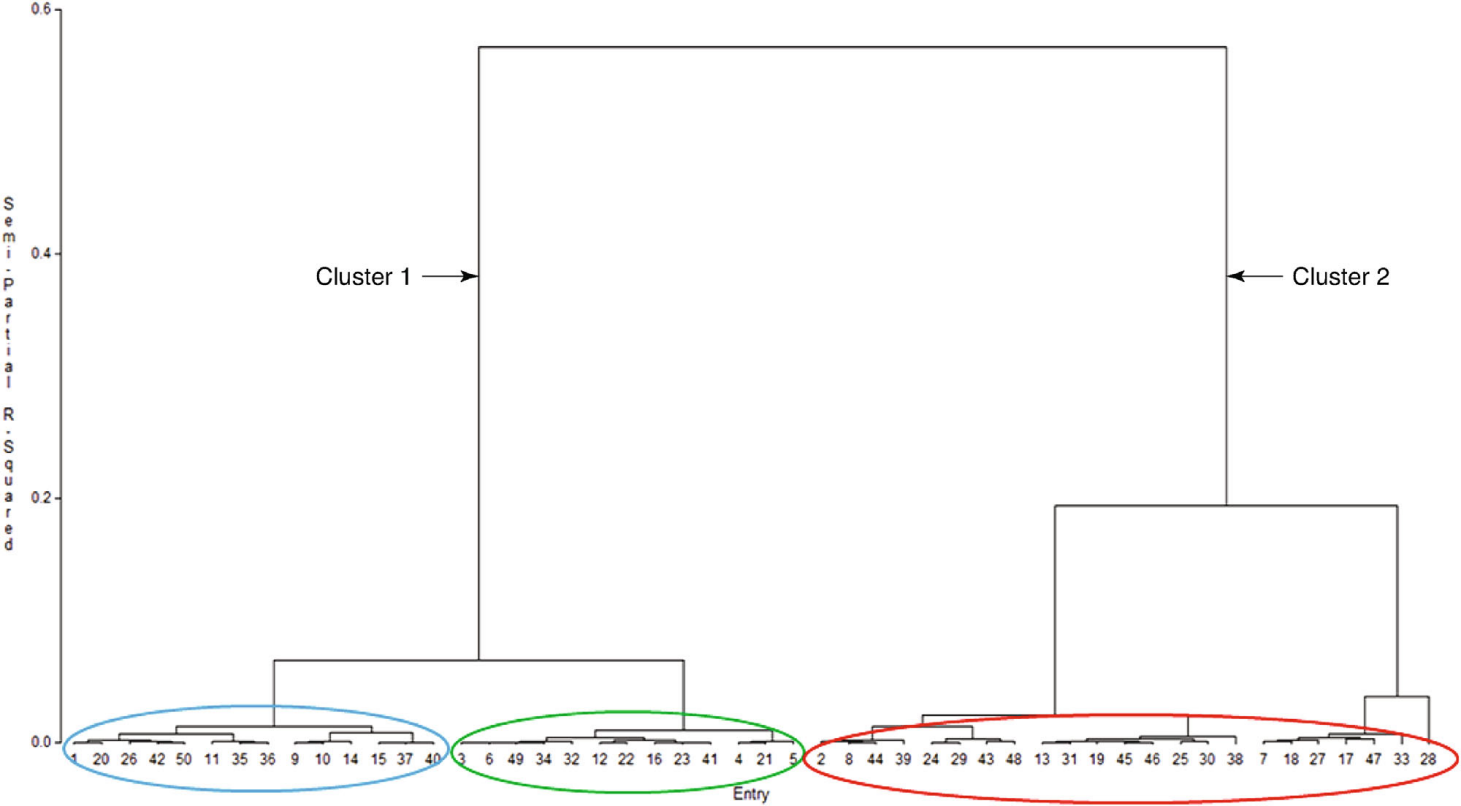
Dendrogram of 50 inbred lines based on three rust scores (Rust 1, Rust 2, and Rust 3) and AUDPC under artificial inoculation with *P. sorghi* for three seasons using Ward's minimum variance method. Blue, green and red ellipses represent lines classified as moderately resistant/resistant, resistant/highly resistant, and moderately susceptible/susceptible/highly susceptible, respectively

Based on Rust 3 severity scores across environments, lines were classified as either highly resistant (HR, score ≤3), resistant (score 3.1–3.9), moderately resistant (MR, score 4–5.5), moderately susceptible (MS, score 5.6–6.5), susceptible (score 6.6–7.5), or highly susceptible (HS, score ≥7.5). Eleven new elite inbred lines and CML395, a widely used line in sub‐Saharan Africa (SSA) were classified as highly resistant, five lines were classified as resistant, while 17 lines were classified as MR (Table 4). Inbred line CML444 that was included in the study as a resistant check was classified as MR. Based on AUPDC no line escaped infection or expressed immunity to CR. Using a combination of the three rust scores and AUDPC, the lines clustered into two clusters according to their response to artificial inoculation with *P. sorghi* (Figure 2). The resistant/moderately resistant (blue ellipse) and highly resistant/resistant (green ellipse) lines formed one cluster while the moderately susceptible, susceptible, and highly susceptible made up the other cluster (red ellipse). The only exception were seven lines (13, 19, 25, 31, 38, 45 and 46) which were classified as MR based on Rust 3 but clustered with the susceptible group.

### 3.3 Consistency of line performance and relationships among rust severity scores and agronomic traits

The consistency of ranking of the inbred lines for their response to CR was assessed using Kendall's coefficient of concordance. The coefficient of concordance (*W*) was moderate for Rust 1 and Rust 3 and high for Rust 2 and AUDPC and was significant (*P* < .0001 or *P* = .001) for all traits (Supplemental Table S4). There were highly significant correlations between the three CR severity parameters, and between CR severity and AUDPC across seasons (Table 5). The AUDPC had a significant correlation with Rust 2 score (*r* = 0.984, *P *< .0001). Gray leaf spot severity was negatively and significantly correlated with all three CR severity parameters and AUDPC. The genetic correlation between CR disease resistance parameters ranged from 0.387 to 0.990 (Table 5). All the three CR disease resistance parameters had a positive and significant genetic correlation with AUDPC (*r* = 0.965–0.987, *P *< .0001). The genetic correlation between the CR disease resistance parameters and GLS and grain yield was negative and significant. Rust 3 showed a negative significant phenotypic correlation with grain yield.

**TABLE 5:** Phenotypic (above diagonal) and genetic (below diagonal) correlation coefficients between BLUPs for common rust (*Puccinia sorghi*) disease rating and AUDPC across three seasons under artificial inoculation and gray leaf spot (GLS) severity and grain yield

	Rust 1	Rust 2	Rust 3	AUDPC	GLS	Grain yield
Rust 1	–	0.876:::	0.770:::	0.893:::	−0.535:::	−0.194 ns
Rust 2	0.987:::	–	0.870:::	0.984:::	−0.591:::	−0.262 ns
Rust 3	0.387:::	0.759:::	–	0.922:::	−0.510:::	−0.293^:^
AUDPC	0.987:::	0.985:::	0.965:::	–	−0.592:::	−0.256 ns
GLS	−0.845:::	−0.678:::	−0.310^:^	−0.626:::	–	−0.113 ns
Grain yield	−0.318^:^	−0.372^::^	−0.516:::	−0.375^::^	−0.099 ns	

^:, ::, :::^Significant at the .05, .01, and .001 probability levels, respectively.

^a^ AUDPC, area under disease progress curve; Rust 1, 2 and 3 are common rust severity scores 21, 28 and 35 days after inoculation, respectively.

ns, not significant.

**FIGURE 3:**
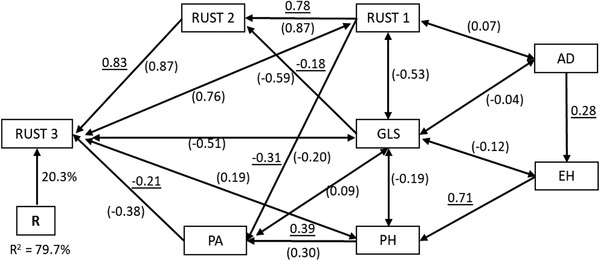
Path analysis model diagram showing causal relationships among CR disease severity parameters, agronomic traits and GLS. The underlined values are direct path coefficients while values in parenthesis are correlation coefficients. Single‐arrowhead line represents a path coefficient while double‐arrowhead line is a correlation coefficient. AD, days to anthesis; EH, ear height; GLS, gray leaf spot; PA, plant aspect; PH, plant height; R, residual effects; Rust 1, 2, and 3 are CR disease severity scores at 21, 28, and 35 days after inoculation, respectively

Spearman rank correlation coefficients for CR disease resistance parameters and AUDPC between seasons were significant (*P *< .0001) and ranged from 0.745–0.886 (Supplemental Table S5). Six of the 12 lines classified as HR using combined data (lines 3, 4, 5, 16, 21, and 32) were consistently ranked in the HR category in each of the three seasons (Supplemental Table S2). Lines 22, 23, 34, 41 and 49 which were also classified as HR were consistent as HR in two of the three seasons. Path analysis using stepwise regression identified Rust 2 and PA as the first‐order traits, which accounted for 79% of the variability in Rust 3 scores under artificial inoculation across three seasons (Figure 3). Of the two traits, Rust 2 had the largest and positive path coefficient (0.83). Rust 1, GLS, and PH were the second‐order traits that contributed to variation in Rust 3 scores, with Rust 1 and GLS contributing indirectly via Rust 2, while PH acted through PA. Rust 1 had the largest indirect effect (0.78) followed by plant height (0.39). Ear height (indirect effect 0.71) and AD (indirect effect 0.28) were the third‐ and fourth‐order traits that contributed to variation in Rust 3 scores through PH and EH, respectively.

### 3.4 Diversity of lines

**FIGURE 4:**
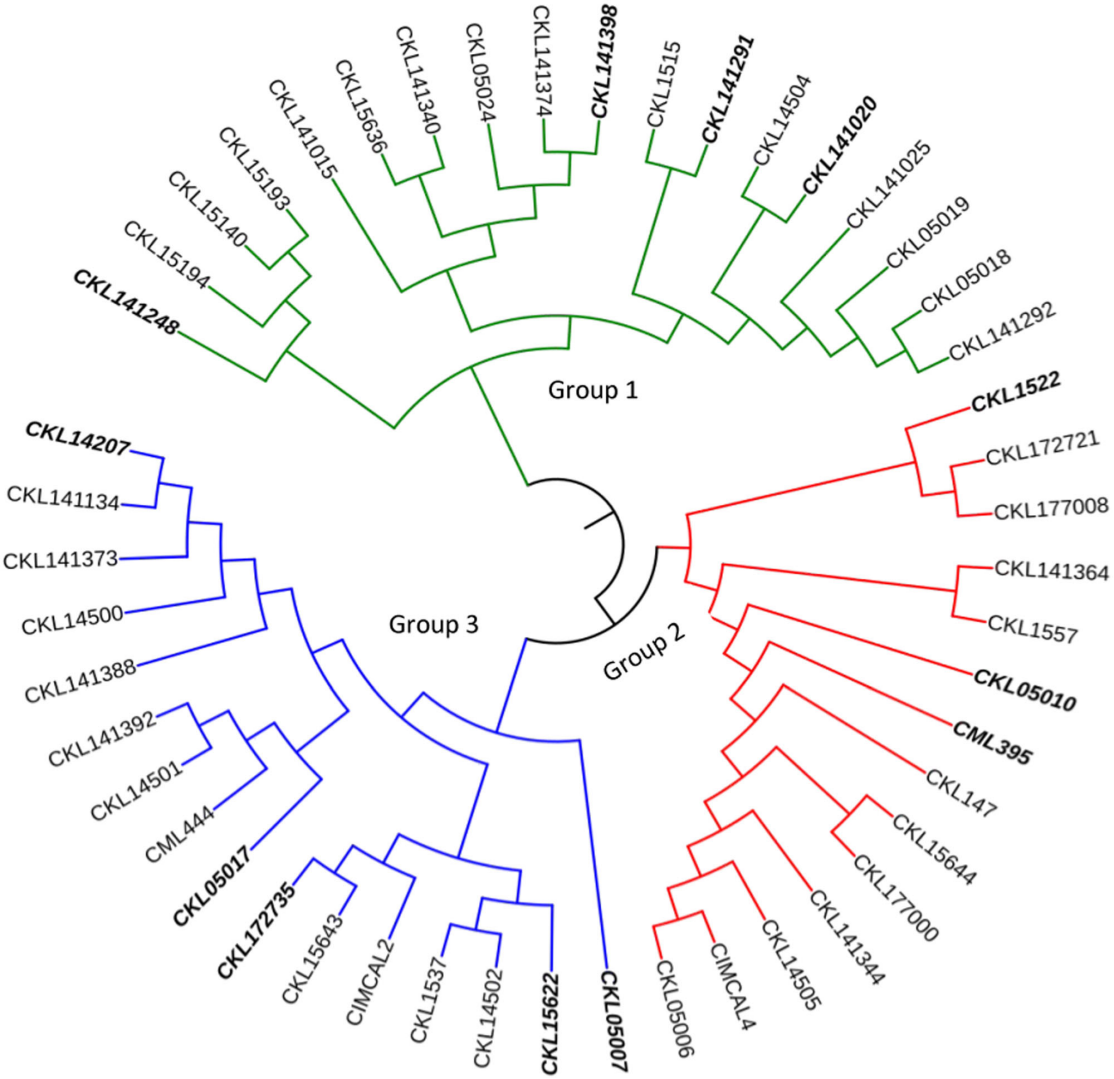
Phylogenetic tree for 50 maize inbred lines based on genetic distance calculated from identity by state matrix using 44,975 DArTseq SNP markers. The clusters have been colored to match the groups from STRUCTURE. The highly resistant inbred lines are shown in bold text

**FIGURE 5:**
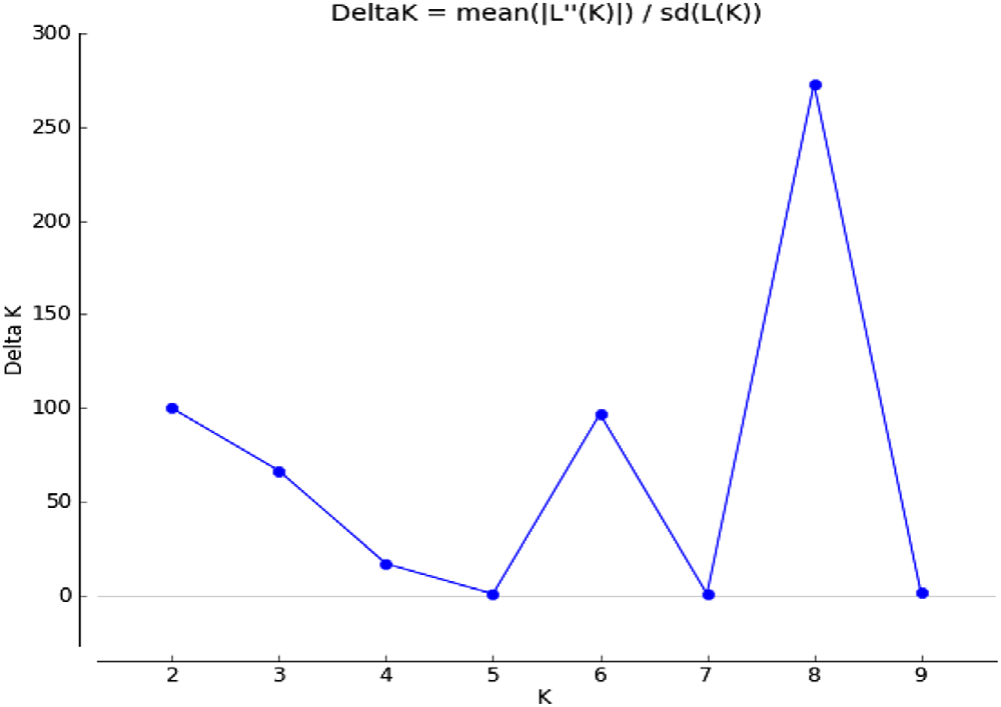
Plot of changes in ΔK value with the number of subpopulations

**FIGURE 6:**
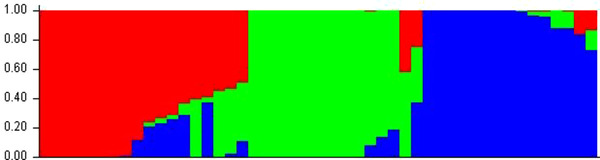
Population structure of the 50 inbred lines at ∆K = 3 based on 44,975 DArTseq SNP markers. Each sub‐population is represented by a different color (1 = red, 2 = green, 3 = blue)

The neighbor‐joining (NJ) tree generated from identity by state matrix was used to understand the genetic diversity among this set maize lines. The dendrogram showed that the 48 lines were grouped into three major groups (Figure 4). The three groups consisted of 18 (37.5%), 14 (29.2%) and 16 (33.3%) inbred lines, respectively. Groups 1, 2 and 3 consisted of three, two and four subgroups, respectively. The three clusters did not show a clear pattern related to pedigree or breeding history. For example, the lines derived from CKL05006 (line 2; group 2) were in both groups 1 (six lines) and 2 (one line). Group 2 had five out of seven lines derived from the drought tolerant La Posta Sequía C7 background. Lines with temperate germplasm were in both groups 2 (three lines) and 3 (one line). Group 3 had four out of five lines with background of germplasm from IITA while the fifth line was in group 2. The lines classified as highly resistant to CR were spread across the three groups with four, three and five lines in groups 1, 2 and 3, respectively. Results from STRUCTURE suggested the presence of either 2, 3, 4, 6 or 8 subgroups among the 48 lines (Figure 5). The ad hoc statistic ΔK sharply increased between K = 6 and K = 8, and less between K = 2 and K = 4. When the results from different K values were compared with pedigree and breeding history, the groups obtained at K = 3 appear to be the best possible number of populations (Figure 6). With K = 3, there was close agreement with the three groups based on NJ clustering. The membership of the lines in the 3 subgroups based on STRUCTURE was 37.5% (group 1) and 31.3% (groups 2 and 3).

## 4 DISCUSSION

Developing adapted maize germplasm with resistance to CR (*Puccinia sorghi*) for the target agro‐ecologies in SSA is an important breeding objective. Common rust has been projected to expand within the region in response to climate change (Ramirez‐Cabral et al., 2017). This study assessed response of diverse maize inbred lines to CR under artificial inoculation for three seasons. Results revealed significant differences among lines for CR, suggesting the presence of sufficient genetic variation that can be utilized in breeding for resistance to CR (Gingera et al., 1994; Kim & Brewbaker, 1977; Zheng et al., 2018). There was seasonal variation in CR severity which may be attributed to differences in temperature and relative humidity during field experimentation across the three seasons. Similar results have been reported for CR and southern leaf rust (Bailey, Wolfgang, Frederiksen, Bockholt, & Smith, 1987; Zheng et al., 2018). However, the AUDPC was consistent across seasons.

The line × environment interaction was not significant for Rust 2 and 3. This result is similar to that reported for lines and single cross hybrids evaluated for resistance to southern rust (Bailey et al., 1987) and for segregating populations evaluated for CR (Gingera et al., 1994). Limited line × environment interaction could be attributed to a longer first pustule appearance after inoculation (Gingera & Davis, 1995). The possible cause of nonsignificant line × environment interaction could not be established using the data collected in this study. The lack of a significant line × environment interaction suggested consistent response of the lines across the seasons for these two scores. The consistency of response of the lines to CR was supported by the significant rank correlation coefficients for the rust scores and AUDPC across seasons. Furthermore, there was consistent performance of lines as revealed by the significant coefficient of concordance. The consistency of performance suggests little or no variation between the rust inoculum across the three seasons and uniformity in inoculation procedures. The lines that were consistently classified as HR in each of the three seasons probably have stable resistance to CR. Identification of stable sources of resistance offers good prospects for control of CR through host‐plant resistance. The AUDPC did not show a significant line × environment interaction mean square, a result similar to that reported by Bailey et al. (1987). The heritability estimates were high for the disease resistance parameters every season and across seasons except for Rust 1, which could indicate consistency of disease pressure in the different seasons. The high heritability estimates for these two parameters suggests that genetic gain from selection for resistance to CR is likely to be high (Falconer & Mackay, 1996).

The inbred lines exhibited good variation in response to inoculation with *P. sorghi*. We identified 12 lines that were highly resistance to CR, and whose response to CR was better than that of the widely used inbred line CML444 which was included as a resistant check. Inbred lines CKL05010, CKL05017, CKL1522 and CKL141398 showed the best response to CR and could be used as donors for CR resistance. The identification of highly resistant lines was based on multiple disease severity ratings and AUDPC, which increases reliability and allows better control of selection decisions for disease resistance (Brown et al., 2001; Carson, 2006). The use of multiple disease ratings to identify disease resistance is useful in screening germplasm (Menkir & Ayodele, 2005; Zheng et al., 2018). Nine of the 12 inbred lines classified as highly resistant are newer inbred lines developed between 2014 and 2017 by combining elite lines from different CIMMYT maize breeding hubs in Kenya, Zimbabwe, Mexico and Colombia, and from IITA. These lines may potentially provide new alleles for resistance to *P. sorghi* and other adaptive traits that can be exploited to develop the new lines with multiple stress tolerance and higher yield potential.

Use of exotic germplasm is important for enriching the gene pool of different breeding programs to enhance genetic gains (Cupertino‐Rodrigues, Dhliwayo, Trachsel, Guo, & San Vicente, 2020; Menkir et al., 2006). Inbred line CKL177000 (line 38) derived from a backcross population between CKL05003 (MR) and an expired Plant Variety Protection Act line PHG39 (PI 600981) known to be susceptible to southern leaf rust expressed moderate resistance to CR. PHG39 has been reported to combine well with some tropical lines and expressed positive general combining ability for grain yield (Cupertino‐Rodrigues et al., 2020). Most of the highly resistant lines identified in this study are resistant/tolerant to NCLB, GLS, and maize streak virus (CIMMYT, unpublished data, 2015) which makes them good candidates for use in biparental populations for line development. In SSA, multiple disease resistance in parental inbred lines is important as there are several foliar and viral diseases some of which occur in combination that must be addressed through breeding (Fininsa & Yuen, 2001; Okori, Adipala, & Kyetere, 1999; Pratt et al., 2003; Vivek et al., 2010).

Resistance to CR is governed by a set of >25 dominant race‐specific *Rp* genes identified in diverse germplasm (Chavan, Gray, & Smith, 2015; Delaney, Webb, & Hulbert, 1998; Groth, Pataky, & Gingera, 1992; Hulbert, 1997; Webb et al., 2002). Limited information is available on which clusters of the *Rp* genes are present in tropical maize germplasm outside of the report by Wilkinson and Hooker (1968). Although host genetic background and the environmental conditions may significantly affect the performance of resistance genes, an investigation to determine the clusters of genes present in tropical germplasm highly resistant, resistant or MR to CR should be considered. A few studies have reported QTLs for rust resistance in tropical maize (Danson, Lagat, Kimani, & Kuria, 2008; Rossi et al., 2020; Zheng et al., 2018). Some of the highly resistant lines identified in this study could be good candidates to develop populations for fine mapping some of the large effect QTLs for resistance to CR (Danson et al., 2008; Rossi et al., 2020; Zheng et al., 2018) and potentially for development of breeder‐ready markers for selection in large breeding populations.

A genetic correlation between two traits may be due to pleiotropy and linkage disequilibrium while a phenotypic correlation is an additive combination of both genetic and environmental correlations. Both phenotypic and genetic correlations among CR scores were positive and strong. Based on this result and high heritability estimates for Rust 2, disease severity recorded 28 days after inoculation can reliably predict CR severity in later phenological stages. The strong positive genetic correlation between the CR resistance parameters suggests that these parameters are controlled by the same set of genes. Strong phenotypic correlations between disease severity ratings taken at multiple intervals have also been reported by Gingera et al. (1994) for CR and by Bailey et al. (1987) for southern rust. The correlation between the CR scores and AUDPC was strong, which suggested that AUDPC is a good measure of resistance. AUDPC does not vary with rate of disease development and should be routinely used for disease resistance screening.

In this study, we recorded a strong negative genetic correlation between CR scores 1 and 2 with GLS, which suggested that greater resistance to CR was associated with reduced resistance to GLS. This underscores the importance of testing lines across multiple biotic stresses and use of a multivariate selection index to select lines with resistance to both CR and GLS. This result is contrary to findings by Olukolu et al. (2016) who reported positive and small genetic correlation between CR and GLS. The difference in results between two studies could be attributed to different germplasm used. Path coefficient analysis was used to understand the important interrelationships among CR resistance parameters and agronomic traits. Path coefficient analysis indicated that Rust 2 had the largest direct path coefficient (0.83) with Rust 3. This suggests that to evaluate resistance to CR in different genotypes under artificial inoculation, disease rating taken 28 days after inoculation is a good indicator of scores 35 days after inoculation. A strong and significant genetic correlation between Rust 2 and Rust 3 (*r* = 0.759, *P *< .0001) further supports this conclusion. Taking CR disease score once 35 days after inoculation can save time and labor. However, disease resistance breeding programs in localities where labor is not expensive, should use AUDPC as the best measure of disease resistance.

Genetic diversity was assessed using identity by state and STRUCTURE in this set of lines. Clustering revealed genetic diversity among the inbred lines as showed by the three distinct clusters generated. This can be attributed to the diverse nature of the bi‐parental populations used to develop the lines which included tropical, subtropical, and temperate sources. Several studies with tropical maize inbred lines have reported diversity which was attributed to the large make‐up of the source germplasm used to develop the lines (Semagn et al., 2012b; Sserumaga et al., 2019; Warburton et al., 2002, 2008; Wu et al., 2016). Furthermore, the variation can be attributed to breeding system, selection, and differences in geographical origin of the source populations. It is worth noting that the new lines with resistance to CR were spread across the three clusters. This suggested diversity among these lines can be exploited to develop new lines through recycling. Since these lines already possess resistance to multiple diseases (foliar and virus diseases), they are potentially good candidates for recycling. For example, highly resistant line 21 (CKL1522, group 2, heterotic group B) could be used as a source of alleles to improve CR resistance in MR line 35 (CKL15636) and MS line 24 (CKL141340) in group 1, and MR line 19 (CKL14502, group 3) that have alleles from several other breeding programs. Similarly, highly resistant line 5 (CKL05017, group 3, heterotic group A) could be used to improve CR resistance in line 9 (resistant, group 2), which is a product of CIMMYT × IITA germplasm. Such bi‐parental crosses between genetically divergent lines will potentially result into combinations of several favorable alleles at different loci leading to new mid‐altitude adapted inbred lines with higher yield potential and multiple disease resistance.

## 5 CONCLUSIONS

This study identified 12 inbred lines highly resistant to CR based on multiple disease severity ratings and AUDPC under artificial inoculation with *Puccinia sorghi* for three seasons. Eight of the 12 highly resistant lines were developed between 2014 and 2017 from different germplasm sources and contain many beneficial alleles for other traits. Assessment of CR resistance in a breeding program should be done using AUDPC which is not affected by environmental conditions. The diversity present among the lines can be exploited by recycling some of the highly resistant genetically distant lines with good genetic potential for important traits to develop new high yielding and multiple disease resistant inbred lines for hybrid development and deployment. The resistant lines could be used develop mapping populations to fine map QTLs for CR resistance. Furthermore, these highly resistant lines can be used to widen genetic diversity in other tropical maize breeding programs.

## AUTHOR CONTRIBUTIONS STATEMENT

JPS planned and performed field experiments and genotyping, analyzed data, and drafted the manuscript; DM developed genetic materials, planned field experiments, analyzed data, drafted and reviewed the manuscript; SOA, EKM, and SGN developed genetic materials and reviewed the manuscript; BMJ and AYB planned experiments and reviewed the manuscript.

## CONFLICT OF INTEREST

The authors declare that there is no conflict of interest.

## Supporting information

Supplemental Table S1. Minimum and maximum temperature and relative humidity for three cropping seasons during which 50 maize inbred lines were evaluated for response to common rust under artificial inoculation with at Namulonge, Uganda, 2017‐2019.
Supplemental Table S2. BLUPs of common rust (*Puccinia sorghi*) disease severity and area under disease progress curve (AUDPC) of 50 tropical maize inbred lines evaluated under artificial inoculation for three seasons, 2017‐2019.
Supplemental Table S3. Summary ANOVA for agronomic traits of 50 inbred lines evaluated under artificial inoculation with *P. sorghi* at Namulonge, Uganda, 2017‐2019.
Supplemental Table S4. Kendall's coefficient of concordance (W) for 50 inbred lines evaluated under artificial inoculation with common rust (*Puccinia sorghi*) for three seasons at Namulonge, Uganda, 2017‐2019.
Supplemental Table S5. Spearman rank correlation coefficients between BLUPs for common rust (*Puccinia sorghi*) disease ratings and AUDPC across three seasons under artificial inoculation and gray leaf spot (GLS) severity.
