# Supplementary material for: Identification and diversity of tropical maize inbred lines with resistance to common rust (Puccinia sorghi Schwein)
Source: Crop Sci. 2020 Nov 11;60(6):2971–89. doi: 10.1002/csc2.20345 (PMC7839556; doi:10.1002/csc2.20345)
Supplement: Supplementary file 1 — Supplemental Table S1. Minimum and maximum temperature and relative humidity for three cropping seasons during which 50 maize inbred lines were evaluated for response to common rust under artificial inoculation with at Namulonge, Uganda, 2017‐2019. Supplemental Table S2. BLUPs of common rust (Puccinia sorghi) disease severity and area under disease progress curve (AUDPC) of 50 tropical maize inbred lines evaluated under artificial inoculation for three seasons, 2017‐2019. Supplemental Table S3. Summary ANOVA for agronomic traits of 50 inbred lines evaluated under artificial inoculation with P. sorghi at Namulonge, Uganda, 2017‐2019. Supplemental Table S4. Kendall's coefficient of concordance (W) for 50 inbred lines evaluated under artificial inoculation with common rust (Puccinia sorghi) for three seasons at Namulonge, Uganda, 2017‐2019. Supplemental Table S5. Spearman rank correlation coefficients between BLUPs for common rust (Puccinia sorghi) disease ratings and AUDPC across three seasons under artificial inoculation and gray leaf spot (GLS) severity. [file CSC2-60-2971-s001.docx]

Supplemental Table 1. Minimum and maximum temperature and relative humidity for three cropping seasons during which 50 maize inbred lines were evaluated for response to common rust under artificial inoculation with at Namulonge, Uganda, 2017-2019.

|  |  | April | May | June | July | August |
| --- | --- | --- | --- | --- | --- | --- |
|  |  | Temperature (^o^C) | | | | |
| 2017 | Minimum | 19.1 | 18.8 | 17.9 | 17.5 | 17.6 |
|  | Maximum | 29.0 | 27.8 | 29.0 | 27.5 | 28.4 |
| 2018 | Minimum | 17.9 | 17.9 | 17.5 | 16.9 | 17.2 |
|  | Maximum | 26.8 | 27.2 | 26.7 | 27.1 | 27.4 |
| 2019 | Minimum | 18.7 | 18.5 | 18.2 | 17.1 | 17.7 |
|  | Maximum | 29.8 | 28.0 | 26.8 | 27.1 | 27.2 |
|  |  | Relative humidity (%) | | | | |
| 2017 | Minimum | 67.1 | 70.4 | 63.2 | 69.6 | 65.4 |
|  | Maximum | 85.6 | 87.2 | 84.4 | 88.6 | 87.4 |
| 2018 | Minimum | 72.6 | 74.7 | 71.1 | 62.3 | 65.8 |
|  | Maximum | 89.4 | 89.6 | 88.8 | 86.3 | 88.6 |
| 2019 | Minimum | 63.0 | 70.0 | 75.1 | 68.4 | 70.9 |
|  | Maximum | 87.2 | 90.3 | 92.4 | 93.2 | 90.8 |

Supplemental Table 2. BLUPs of common rust (*Puccinia sorghi*) disease severity and area under disease progress curve (AUDPC) of 50 tropical maize inbred lines evaluated under artificial inoculation for three seasons, 2017-2019.

|  |  | 2017 | | | |  | 2018 | | | |  | 2019 | | |
| --- | --- | --- | --- | --- | --- | --- | --- | --- | --- | --- | --- | --- | --- | --- |
|  |  | Rust severity | | |  |  | Rust severity | | |  |  | Rust severity | |  |
| Line | Name | 1 | 2 | 3 | AUDPC^†^ |  | 1 | 2 | 3 | AUDPC |  | 1 | 2 | AUDPC |
| 1 | CKL05003 | 1.0 | 2.0 | 3.9 | 31.6 |  | 1.0 | 1.8 | 4.8 | 32.9 |  | 1.1 | 2.3 | 20.4 |
| 2 | CKL05006 | 2.0 | 2.0 | 4.8 | 37.6 |  | 1.0 | 2.3 | 7.2 | 45.6 |  | 1.6 | 3.7 | 31.2 |
| 3 | CKL05007 | 1.0 | 2.0 | 2.9 | 27.7 |  | 1.5 | 1.3 | 2.3 | 19.7 |  | 1.6 | 2.9 | 25.3 |
| 4 | CKL05010 | 1.0 | 1.0 | 2.1 | 18.5 |  | 1.0 | 1.5 | 1.4 | 22.3 |  | 1.0 | 2.2 | 17.7 |
| 5 | CKL05017 | 1.0 | 1.5 | 2.4 | 22.1 |  | 1.0 | 1.0 | 1.2 | 11.3 |  | 1.7 | 1.9 | 20.0 |
| 6 | CKL05018 | 1.5 | 2.0 | 3.0 | 30.3 |  | 1.5 | 2.2 | 3.3 | 31.4 |  | 1.0 | 2.2 | 15.9 |
| 7 | CKL05019 | 1.5 | 3.5 | 8.1 | 58.6 |  | 1.5 | 4.1 | 7.3 | 58.9 |  | 4.0 | 6.4 | 58.8 |
| 8 | CKL05024 | 1.5 | 2.5 | 5.7 | 41.8 |  | 1.0 | 3.0 | 8.4 | 39.9 |  | 2.1 | 4.1 | 36.8 |
| 9 | CKL147 | 1.5 | 2.0 | 2.6 | 28.8 |  | 1.5 | 2.3 | 4.3 | 36.9 |  | 1.4 | 3.7 | 31.5 |
| 10 | CKL14504 | 1.1 | 2.0 | 2.5 | 26.3 |  | 1.0 | 1.6 | 5.2 | 35.2 |  | 2.1 | 3.7 | 32.6 |
| 11 | CKL14505 | 1.4 | 2.5 | 2.7 | 31.2 |  | 1.0 | 1.9 | 7.0 | 42.7 |  | 1.0 | 5.7 | 41.4 |
| 12 | CKL141015 | 1.0 | 2.0 | 3.7 | 30.2 |  | 1.0 | 1.1 | 3.2 | 21.9 |  | 1.0 | 1.7 | 14.5 |
| 13 | CKL141025 | 1.6 | 2.0 | 4.6 | 36.2 |  | 1.0 | 2.8 | 4.3 | 41.8 |  | 2.2 | 3.7 | 34.1 |
| 14 | CKL141291 | 2.0 | 2.0 | 2.4 | 29.1 |  | 1.5 | 1.7 | 3.2 | 27.9 |  | 1.7 | 4.1 | 37.6 |
| 15 | CKL141292 | 2.0 | 2.0 | 3.2 | 32.0 |  | 1.5 | 1.8 | 7.6 | 42.6 |  | 1.4 | 3.0 | 26.4 |
| 16 | CKL141020 | 1.0 | 1.5 | 2.0 | 20.6 |  | 1.5 | 1.6 | 2.5 | 23.8 |  | 1.1 | 2.9 | 24.2 |
| 17 | CKL14500 | 2.0 | 4.0 | 6.6 | 58.3 |  | 2.5 | 4.6 | 6.7 | 64.8 |  | 3.8 | 5.3 | 50.1 |
| 18 | CKL14501 | 2.5 | 3.5 | 5.9 | 54.9 |  | 2.0 | 4.2 | 7.8 | 64.7 |  | 3.3 | 5.1 | 48.4 |
| 19 | CKL14502 | 2.0 | 3.0 | 3.2 | 39.1 |  | 1.5 | 3.3 | 5.8 | 47.5 |  | 2.0 | 4.4 | 37.1 |
| 20 | CKL1515 | 1.5 | 2.0 | 2.5 | 28.1 |  | 1.0 | 1.8 | 4.9 | 32.8 |  | 1.0 | 3.0 | 22.3 |
| 21 | CKL1522 | 1.1 | 1.9 | 2.0 | 25.3 |  | 1.0 | 1.4 | 1.4 | 18.6 |  | 1.0 | 1.3 | 10.4 |
| 22 | CKL14207 | 1.0 | 1.5 | 2.0 | 20.7 |  | 1.0 | 1.2 | 3.7 | 25.2 |  | 1.1 | 2.5 | 21.7 |
| 23 | CKL141248 | 1.0 | 1.5 | 2.0 | 20.7 |  | 1.0 | 2.4 | 4.0 | 33.7 |  | 1.0 | 2.7 | 20.0 |
| 24 | CKL141340 | 1.0 | 3.5 | 6.5 | 49.9 |  | 1.0 | 2.9 | 5.0 | 42.7 |  | 3.3 | 5.5 | 51.4 |
| 25 | CKL141340 | 1.5 | 3.5 | 5.3 | 47.9 |  | 1.0 | 1.9 | 5.1 | 34.3 |  | 3.6 | 4.8 | 46.2 |
| 26 | CKL141344 | 1.0 | 2.0 | 3.3 | 28.7 |  | 1.0 | 2.9 | 5.9 | 45.2 |  | 1.2 | 3.0 | 25.5 |
| 27 | CKL141364 | 2.0 | 3.5 | 7.2 | 56.3 |  | 1.5 | 3.2 | 8.0 | 43.1 |  | 3.1 | 5.5 | 49.8 |
| 28 | CKL141373 | 2.9 | 5.5 | 8.9 | 79.4 |  | 2.0 | 6.6 | 8.3 | 82.2 |  | 4.0 | 7.6 | 66.1 |
| 29 | CKL141374 | 1.1 | 2.5 | 3.6 | 34.1 |  | 2.0 | 4.8 | 8.0 | 66.2 |  | 2.1 | 4.9 | 41.1 |
| 30 | CKL141388 | 1.5 | 3.0 | 5.4 | 44.9 |  | 1.5 | 2.3 | 5.5 | 42.8 |  | 2.6 | 4.1 | 38.2 |
| 31 | CKL141392 | 2.0 | 2.5 | 5.1 | 42.7 |  | 1.5 | 1.9 | 4.2 | 32.5 |  | 1.7 | 4.4 | 35.8 |
| 32 | CKL141398 | 1.5 | 2.0 | 2.4 | 27.7 |  | 1.0 | 1.3 | 1.9 | 19.7 |  | 1.4 | 2.0 | 18.1 |
| 33 | CKL141134 | 2.5 | 5.0 | 8.9 | 74.4 |  | 1.0 | 3.7 | 8.0 | 56.9 |  | 4.3 | 6.9 | 62.9 |
| 34 | CKL15622 | 1.1 | 1.5 | 2.6 | 23.7 |  | 1.0 | 1.1 | 3.4 | 23.9 |  | 2.2 | 2.9 | 28.8 |
| 35 | CKL15636 | 1.6 | 3.0 | 4.7 | 44.0 |  | 1.0 | 2.2 | 4.0 | 34.0 |  | 1.5 | 3.5 | 29.8 |
| 36 | CKL15643 | 1.0 | 2.0 | 3.9 | 30.8 |  | 1.5 | 2.6 | 4.8 | 40.8 |  | 1.5 | 3.8 | 32.0 |
| 37 | CKL15644 | 1.0 | 2.0 | 4.9 | 34.2 |  | 1.5 | 2.2 | 5.4 | 39.1 |  | 2.6 | 3.9 | 36.4 |
| 38 | CKL177000 | 2.0 | 2.5 | 3.0 | 34.3 |  | 2.0 | 2.4 | 5.2 | 39.9 |  | 2.7 | 4.5 | 40.7 |
| 39 | CKL172721 | 1.0 | 3.0 | 6.9 | 48.0 |  | 1.5 | 3.1 | 8.4 | 57.2 |  | 1.9 | 3.9 | 33.8 |
| 40 | CKL177008 | 2.0 | 2.5 | 4.1 | 39.0 |  | 1.0 | 1.0 | 6.8 | 15.9 |  | 2.1 | 4.1 | 36.2 |
| 41 | CKL172735 | 1.0 | 2.0 | 2.6 | 27.1 |  | 1.0 | 2.2 | 2.9 | 29.5 |  | 1.2 | 3.1 | 27.1 |
| 42 | CKL1557 | 1.5 | 2.0 | 2.7 | 28.9 |  | 1.0 | 2.1 | 5.5 | 35.1 |  | 1.1 | 2.7 | 23.0 |
| 43 | CKL15140 | 1.4 | 3.0 | 7.7 | 52.0 |  | 1.5 | 2.4 | 5.9 | 42.3 |  | 3.4 | 5.4 | 49.8 |
| 44 | CKL15193 | 1.5 | 3.0 | 5.7 | 46.0 |  | 1.5 | 3.1 | 6.7 | 50.2 |  | 1.6 | 3.1 | 27.3 |
| 45 | CKL15194 | 1.1 | 2.5 | 4.4 | 37.1 |  | 1.0 | 2.5 | 5.2 | 36.3 |  | 2.9 | 4.6 | 43.3 |
| 46 | CKL1537 | 1.4 | 3.0 | 5.4 | 44.5 |  | 2.0 | 3.3 | 4.6 | 45.5 |  | 1.5 | 3.7 | 30.8 |
| 47 | CIMCAL2 | 2.0 | 3.5 | 5.3 | 49.7 |  | 2.0 | 3.8 | 7.2 | 58.9 |  | 3.4 | 5.0 | 46.6 |
| 48 | CIMCAL4 | 1.0 | 3.0 | 6.1 | 45.9 |  | 1.5 | 3.2 | 7.8 | 57.0 |  | 3.3 | 5.2 | 48.4 |
| 49 | CML395 | 1.0 | 1.5 | 3.2 | 25.2 |  | 1.0 | 1.7 | 2.1 | 25.2 |  | 1.7 | 2.8 | 25.4 |
| 50 | CML444 | 1.0 | 2.0 | 3.3 | 28.9 |  | 1.5 | 2.2 | 4.5 | 36.5 |  | 1.5 | 3.6 | 30.6 |
| LSD |  | 0.9 | 1.3 | 2.5 | 16.7 |  | 1.0 | 1.3 | 2.2 | 19.3 |  | 1.5 | 1.1 | 10.3 |
| Genotype |  | * | ** | *** | *** |  | ns | *** | *** | *** |  | *** | *** | *** |
| Heritability |  | 0.52 | 0.72 | 0.77 | 0.80 |  | 0.22 | 0.83 | 0.86 | 0.79 |  | 0.73 | 0.91 | 0.92 |

*,**,*** Significant at the 0.05, 0.01, and 0.001 probability levels, respectively.

^†^AUDPC, area under disease progress curve

Supplemental Table 3. Summary ANOVA for agronomic traits of 50 inbred lines evaluated under artificial inoculation with *P. sorghi* at Namulonge, Uganda, 2017-2019.

| Year | Source | df | GY | AD | PH^†^ | EH^†^ | PA^†^ | GLS |
| --- | --- | --- | --- | --- | --- | --- | --- | --- |
| 2017 | Line | 49 | ** | ** | ns | * | ns | ** |
|  | Block(Rep) | 19 | *** | ns | ns | ** | ns | ns |
|  |  |  |  |  |  |  |  |  |
| 2018 | Line | 49 | *** | *** | - | - | ns | *** |
|  | Block(Rep) | 19 | *** | ** | - | - | ns | ns |
|  |  |  |  |  |  |  |  |  |
| 2019 | Line | 49 | ns | *** | *** | *** | - | ns |
|  | Block(Rep) | 19 | ns | ns | ** | *** | - | ns |
|  |  |  |  |  |  |  |  |  |
| Across | Environment (E) | 2 | *** | *** | *** | ** | ns | *** |
|  | Block(E × Rep) | 57 | *** | ** | ** | *** | ns | ns |
|  | Line | 49 | *** | *** | *** | ** | ns | *** |
|  | Line × E | 98 | ns | ** | ns | ns | ns | * |

*,**,*** Significant at the 0.05, 0.01, and 0.001 probability levels, respectively.

^†^degrees of freedom were 1, 38, and 49 for environment, block(environment × rep) and line × environment, respectively.

Supplemental Table 4. Kendall’s coefficient of concordance (W) for 50 inbred lines evaluated under artificial inoculation with common rust (*Puccinia sorghi*) for three seasons at Namulonge, Uganda, 2017-2019.

| Trait | Number of seasons | W | Prob. |
| --- | --- | --- | --- |
| Rust 1 | 3 | 0.580 | <0.0001 |
| Rust 2 | 3 | 0.828 | <0.0001 |
| Rust 3 | 2 | 0.572 | 0.0010 |
| Area under disease progress curve | 3 | 0.842 | <0.0001 |

Supplemental Table 5. Spearman rank correlation coefficients between BLUPs for common rust (*Puccinia sorghi*) disease ratings and AUDPC across three seasons under artificial inoculation and gray leaf spot (GLS) severity.

|  | Rust 1 | Rust 2 | Rust 3 | AUDPC | GLS |
| --- | --- | --- | --- | --- | --- |
| Rust 1 | - | 0.838*** | 0.770*** | 0.745*** | -0.446*** |
| Rust 2 |  | - | 0.886*** | 0.863*** | -0.570*** |
| Rust 3 |  |  | - | 0.846*** | -0.486*** |
| AUDPC |  |  |  | - | -0.684*** |
| GLS |  |  |  |  | - |

*** Significant at the 0.001 probability level.

^†^AUDPC, area under disease progress curve; Rust 1, 2 and 3 are common rust severity scores 21, 28 and 35 days after inoculation, respectively.
